# Functional magnetic resonance imaging of the trail-making test in older adults

**DOI:** 10.1371/journal.pone.0232469

**Published:** 2020-05-12

**Authors:** Natasha Talwar, Nathan W. Churchill, Megan A. Hird, Fred Tam, Simon J. Graham, Tom A. Schweizer

**Affiliations:** 1 Keenan Research Centre for Biomedical Science, St. Michael's Hospital, Toronto, Canada; 2 Physical Sciences Platform, Sunnybrook Research Institute, Toronto, Canada; 3 Department of Medical Biophysics, Faculty of Medicine, University of Toronto, Toronto, Canada; 4 Division of Neurosurgery, St. Michael’s Hospital, Toronto, Canada; Universidad Nacional Autonoma de Mexico, MEXICO

## Abstract

The trail-making test (TMT) is a popular neuropsychological test, which is used extensively to measure cognitive impairment associated with neurodegenerative disorders in older adults. Behavioural performance on the TMT has been investigated in older populations, but there is limited research on task-related brain activity in older adults. The current study administered a naturalistic version of the TMT to a healthy older-aged population in an MRI environment using a novel, MRI-compatible tablet. Functional MRI was conducted during task completion, allowing characterization of the brain activity associated with the TMT. Performance on the TMT was evaluated using number of errors and seconds per completion of each link. Results are reported for 36 cognitively healthy older adults between the ages of 52 and 85. Task-related activation was observed in extensive regions of the bilateral frontal, parietal, temporal and occipital lobes as well as key motor areas. Increased age was associated with reduced brain activity and worse task performance. Specifically, older age was correlated with decreased task-related activity in the bilateral occipital, temporal and parietal lobes. These results suggest that healthy older aging significantly affects brain function during the TMT, which consequently may result in performance decrements. The current study reveals the brain activation patterns underlying TMT performance in a healthy older aging population, which functions as an important, clinically-relevant control to compare to pathological aging in future investigations.

## Introduction

The trail-making test (TMT) is a pen-and-paper neuropsychological test that is used extensively in research and clinical settings. The TMT consists of two different conditions: TMT-A and TMT-B, each to be performed as quickly as possible while maintaining accuracy, and without removing the tip of the pen from the page[1]. For TMT-A, individuals must connect 25 randomly distributed encircled numbers from 1 to 25 in ascending order (*i*.*e*. 1-2-3-…). For TMT-B, individuals must connect numbers and letters in alternating, ascending order (*i*.*e*. 1-A-2-B-3-C…). The task conditions are scored using metrics that relate to completion times and the number of errors committed. Successful performance of the TMT requires the engagement of numerous cognitive elements including visual search, attention, processing speed, task switching, cognitive flexibility and executive function [1–3].

The sensitivity of the TMT to dysfunction in many cognitive domains has led to its widespread use as a clinical tool to measure cognitive impairment associated with various neurological and psychiatric conditions [4–12]. For example, the TMT is used in clinical settings to screen for neurodegenerative disease in older adults, such as Alzheimer’s Disease (AD) [4,5,7,9–11]. Much of the research investigating the TMT has focused on comparing different performance metrics and measuring changes in TMT performance for elderly and cognitively impaired populations [7,9–11,13,14]. Prior research has established that TMT behavioural performance is significantly impacted by healthy aging, with older adults exhibiting longer task completion times [13–16], likely arising from age-related decline in cognitive domains such as attention, processing speed and mental flexibility [15,16].

At present, however, there are insufficient functional neuroimaging data available for the TMT to increase our scientific understanding beyond this rather general statement. One promising tool for evaluating the neural mechanisms underpinning TMT performance is functional magnetic resonance imaging (fMRI), which measures task-related brain activity based on the blood-oxygenation-level-dependent (BOLD) effect of neurovascular coupling [17]. The fMRI method enables non-invasive measurement of activity throughout the brain volume at spatial and temporal resolutions of millimetres and seconds, respectively. Early fMRI studies of TMT performance involved highly modified versions of the task, with the drawing component replaced by a verbal response or a button-press response [18–20]. Although these studies provided insight into some of the cognitive elements that are required for successful TMT performance, use of a simplified response method meant that it was impossible to characterize the specific brain regions involved in sensation, cognition and action accurately and in their entirety[18–20].

To overcome this limitation and study the TMT in more detail, specialized MRI-compatible technology was developed that facilitated drawing responses (and writing responses) during fMRI. This technology started with a fiber-optic MRI compatible writing device [21] and progressed to a computerized MRI-compatible tablet system with a touch-sensitive screen [22]. Early prototypes of the tablet system were used to deliver simplified versions of the TMT during fMRI [21–23]. However, participants were unable to see their hand, the tablet or the stylus while they were lying supine in the magnet bore engaged in task performance, resulting in their TMT responses relying heavily on proprioception. In the clinical setting, the patient or research participant sits upright with full visualization of the page and their writing movements. The reliance on proprioceptive input sparked further development of the tablet technology to include a video camera and augmented reality display to provide real-time visual feedback of hand position during task completion [24]. Recently, this new tablet prototype was validated as an effective, naturalistic method for administering the TMT and brain activity was investigated during TMT performance in a cohort of healthy young adults [25].

Although functional neuroimaging of TMT performance in healthy young adults is an important first step [18–21,25,26], to our knowledge, fMRI studies of the task have yet to be performed in older individuals–the population where the TMT is predominantly administered in the clinic. The cognitive changes that occur during healthy aging are well known to be accompanied by changes in brain activity, which can present on average as decreased activity associated with functional decline as well as increased activity associated with neural compensation mechanisms [27,28]. Consequently, it is necessary to study the neural correlates of TMT performance in healthy older adults from a basic science perspective, to better understand the effects of cognitive aging, as well as to provide initial normative data prior to conducting clinically-focused fMRI studies of the TMT in elderly patient populations. The present fMRI study addresses these goals by studying the brain and behavioural relationships generated by administering TMT-A and TMT-B to healthy adults over the age of 50, using the enhanced tablet system. It is hypothesized that both task conditions (TMT-A and TMT-B) will recruit extensive brain areas in the bilateral frontal and parietal lobes across the older aging group, consistent with results found in previous fMRI studies with younger populations. Based on prior literature examining TMT performance in healthy older adults [13–16], it is hypothesized that there will be subtle behavioural performance declines in in-scanner TMT performance as represented by slower task completion times, with a corresponding reduction in amplitude and spatial extent of activation within TMT-related brain areas. Age-related declines in activation are further hypothesized to be predominantly in frontal areas involved in TMT performance. Frontal regions, which are essential for controlling cognitive processes involved in the TMT, are susceptible to the effects of age and, therefore, are a likely neural correlate of worsened task performance observed in older adults.

## Materials and methods

### Participants

Thirty-seven (*n* = 37) healthy participants between the ages of 52 and 85 were recruited from the local community into this study. The participants were all screened for history of any neurological or psychiatric condition, neurological incident or substance abuse. The participants were all right handed as determined by the Edinburgh Handedness Inventory [29] and were fluent in English. The study was approved by the Research Ethics Board at St. Michael’s Hospital according to the principles of the Declaration of Helsinki. Participants provided their free and informed consent prior to entering the study.

### Neurocognitive assessment

The Montreal Cognitive Assessment (MoCA) [30] was used to evaluate the cognitive abilities of the participants prior to fMRI. The MoCA score was used to determine whether there were any subjects with significant cognitive impairments. The inclusion criterion was that all participants were to have a MoCA score within 2.5 standard deviations of the group mean. One subject was identified as an outlier on this basis (MoCA score = 15, p ~ 0.01, Normal distribution, 2-tailed) and was not included in any analyses. Participants also completed one sample trial and one full trial of both TMT-A and TMT-B on paper, as the test is normally administered. The paper version of the TMT was administered to investigate convergent validity with the tablet-based TMT. Participants were instructed to work as quickly as they could without making mistakes and not to lift the pen from the paper. The number of errors, number of pen lifts and total time to complete the task was recorded for both the sample and full trials. No time limitation was imposed on performing TMT-A or TMT-B.

### Magnetic resonance imaging

Participants were imaged using the St. Michael’s Hospital Research 3.0 Tesla MRI system (Magnetom Skyra, Siemens Healthineers, Erlangen, Germany), with the standard 20-channel head coil. The structural imaging was performed using the T1-weighted Magnetization Prepared Rapid Acquisition Gradient Echo method (MPRAGE: inversion time (T1)/echo time (TE)/repetition time (TR) = 1090/3.55/2300 ms, flip angle (FA) = 80, bandwidth (BW) = 200 Hz/pixel, sagittal orientation with field of view (FOV) = 240x240 mm, 256x256 matrix, 192 slices, voxels = 0.9 mm x 0.9 mm x 0.9 mm). The functional MRI data were acquired during completion of the TMT using multi-slice T2*-weighted echo planar imaging (EPI: TE/TR = 30/2000 ms, FA = 700, BW = 2298 Hz/px, oblique-axial slices, slices interleaved ascending, with FOV = 200x200 mm, 64x64 matrix, 32 slices with 4.0 mm thickness and 0.5 mm gap, voxels = 3.125 mm x 3.125 mm x 4.5 mm).

### MRI-compatible tablet system

**Fig 1:**
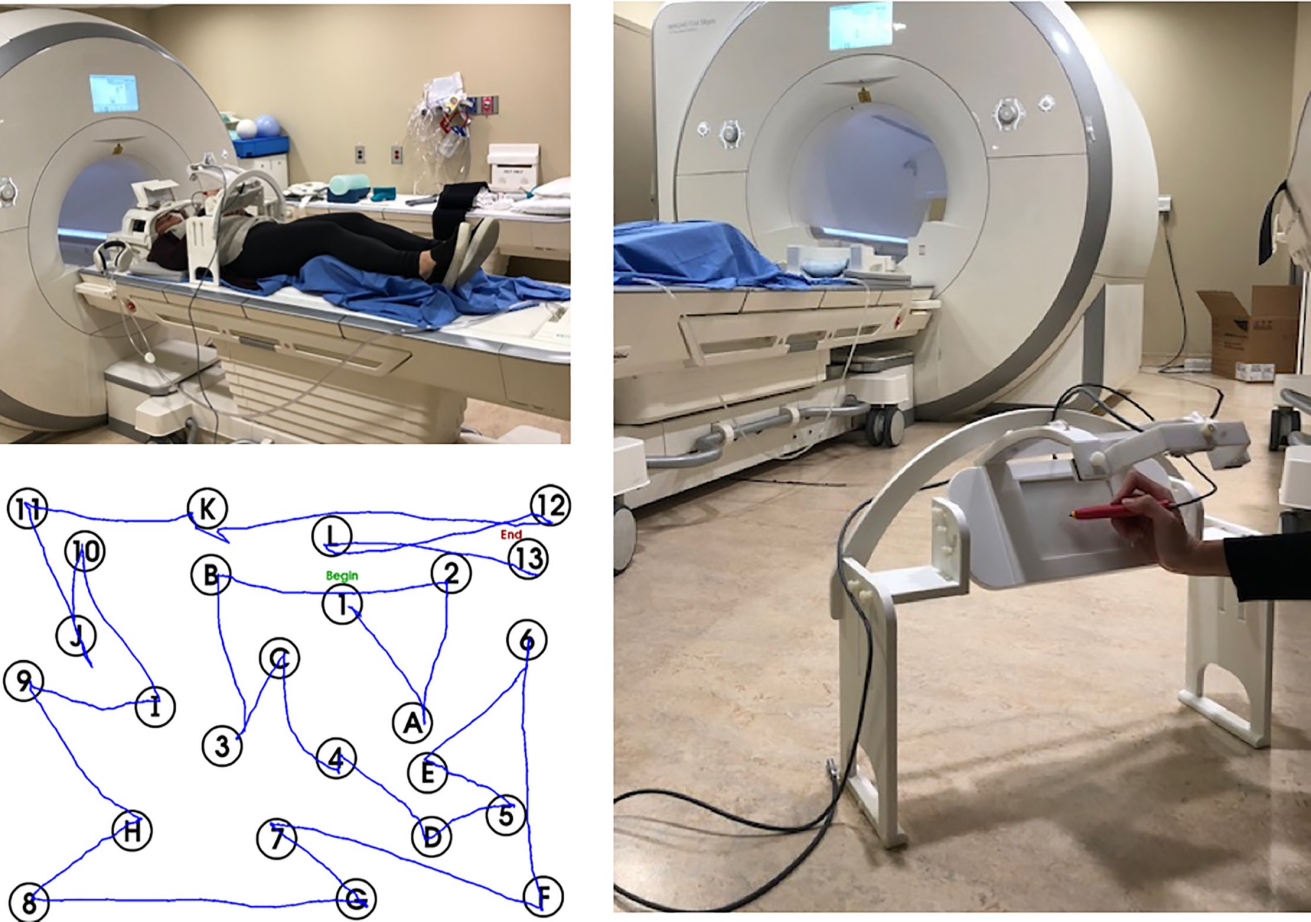
Images of the tablet system set-up. (A) The participant lies supine on the MRI table before entering the magnet, with the tablet mounted at the waist using an adjustable stand. The head coil for receiving fMRI signals includes a mirror to view the task in an augmented reality environment on a display screen using an fMRI-compatible projector (not shown). (B) The tablet mount includes an fMRI-compatible video camera for a top-down view of tablet interactions. (C) Image results of the participant completing the TMT-B task on the tablet. TMT = Trail Making Test. fMRI = functional magnetic resonance imaging.

The MRI-compatible tablet system parallels real-world pen and paper settings by using a touch screen and stylus in conjunction with an augmented reality system to provide real-time visual feedback of hand and stylus position [24]. The tablet and camera were mounted on an adjustable stand, which was placed over the waist of the participant during the imaging session. A mirror was placed on the top of the head coil and angled towards a screen, on which the task and augmented reality environment were displayed using an MRI-compatible projector (Avotec, Stuart, FI). Pads were placed underneath the elbows of the participant to provide comfort and limit movement during task performance. MRI-compatible prescription glasses (MediGlasses for fMRI, Cambridge Research Systems, Kent, UK) were used for participants with corrected vision. The experimental set-up is shown in Fig 1.

### fMRI of the TMT

**Fig 2:**
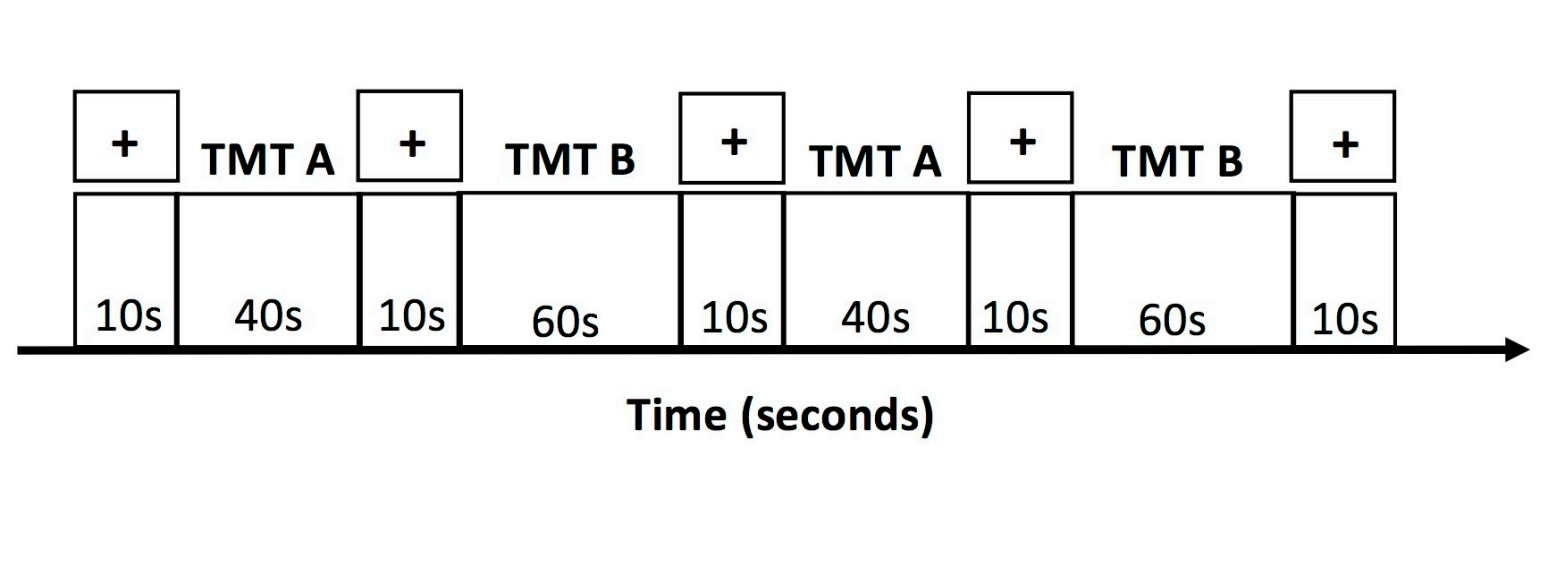
Task design for one run of fMRI time series data collection in this study. TMT = Trail Making Test.

The tablet-based TMT was developed and presented using E-Prime Version 2.0 (Psychology Software Tools, Sharpsburg, PA). Outside of the MRI system, participants were familiarized with the equipment by completing a training session with the tablet and by viewing the set-up in the magnet room. Once in the magnet bore, participants completed additional tablet training to become comfortable with the set-up by performing simple tasks such as writing their name or drawing a house. Participants subsequently completed the tablet-based TMT according to the timing diagram shown in Fig 2, after structural MRI was performed. Two “runs” of fMRI time series data were collected, with each run consisting of TMT-A (40 s) and TMT-B (60 s) interleaved with a visual fixation condition (10 s). Two “trials” of each condition were performed within each run. At the beginning of each run, the participants received on-screen instructions on how to perform each task. Four different iterations with pseudorandom patterns of numbers and letters for both the TMT-A and TMT-B were used overall, two in each run. Both versions of the TMT had 25 targets to link in each iteration. Specifically all trials of TMT-A had 25 numbers (1–25) and all trials of TMT-B had 13 numbers (1–13) and 12 letters (A-L). For the fixation task, participants were asked to fixate their eyes on the center of a black cross on a white background. This version of the tablet-based TMT has been used previously in research and has been validated as a realistic replication of the paper-based TMT [22,25].

### Data analysis

#### Analysis of behavioural measures

The traditional paper-based TMT delivery adopts the completion time as a key measure of performance. However, as is typical of a “block-design” fMRI experiment, brain activity associated with tablet-based TMT delivery was assessed for a fixed time duration. Based on previous tablet-based fMRI of the TMT in young adults [25], it was predicted that some participants would not complete TMT-A and/or TMT-B in the allotted time duration. Given that this precluded measurement of completion time across all participants, a different normalized metric was adopted: the seconds per link (SPL). The SPL value was calculated for both paper-based and tablet-based delivery by dividing the total time spent performing one part of the TMT (*i*.*e*. TMT-A or TMT-B) by the number of correct links completed during that time. Paper-based and tablet-based TMT performance was also compared using a secondary measure, the number of errors (N_E_). The N_E_ value included both self-corrected and uncorrected errors that occurred during the allotted time duration. Given that errors were rare (see Results), no normalization was incorporated into the comparison.

In preliminary behavioural analysis, the effects of trial on SPL were evaluated using a 1-way repeated measures ANOVA, where a significant effect was found (F = 3.94, p = 0.009). Post-hoc statistical testing using Tukey’s test determined that the first tablet-based trial of both TMT-A and TMT-B for run 1 was significantly different from the rest of the tablet-based trials (Trial 2–1, *p* = 0.03, Trial 3–1, *p* = 0.02, Trial 4–1, *p* = 0.78), whereas there was no significant difference between the remaining trials (Trial 3–2, *p* = 1.00, Trial 4–2, *p* = 0.26, Trial 4–3, *p* = 0.23). During the first trial, participants may have been acclimatizing to the fMRI environment and performance of the tablet-based TMT, leading to slower initial performance. The behavioural measures were averaged for each participant across the other three trials of TMT-A and three trials of TMT-B, to improve the statistical power to detect significant behaviour and brain activation effects. To measure the effect of both versions of the task (TMT(A+B)), the behavioural measures were averaged across all six trials of both TMT types. To isolate the effect of TMT-B, the behavioural results for TMT-A were subtracted from the results of TMT-B for each performance measure (TMT-(B-A)) in analogous fashion. Average task performance was correlated with age using a Spearman’s rank correlation. To provide convergent validity of the tablet-based TMT, the SPL for the tablet-based TMT-A, TMT-B and TMT-(B-A) were correlated with the results from the paper version using a Spearman’s rank correlation. Additionally, the SPL for TMT-A, TMT-B and TMT-(B-A) were compared between the two versions of the task (i.e. paper and tablet) using a paired Mann-Whitney U test.

#### fMRI preprocessing and analysis

The fMRI data and structural scans were visually inspected for any gross abnormalities. A hybrid pipeline, which includes tools from the Analysis of Functional Neuroimages (AFNI; https://afni.nimh.nih.gov) package [31], the FMRIB Software Library (FSL; https://www.fmrib.ox.ac.uk/fsl) package [32] and algorithms established in the laboratory, was used for preprocessing and analysis of the imaging data. The preprocessing pipeline included the following sequence of steps: rigid-body motion correction (AFNI *3dvolreg*), slice-timing correction (AFNI *3dTshift*), removal of outlier scan volumes via SPIKECOR [33], spatial smoothing with an isotropic Gaussian kernel of 6 mm full width at half maximum (AFNI *3dmerge*), followed by regression of motion parameters as well as linear and quadratic trends. PHYCAA+ [34] was used to perform data-driven down-weighting of signal from vascular and ventricular regions, followed by further noise reduction using white matter signal regression from seed regions of interest placed in the left and right corona radiata (average cluster size = 1052 mm^3^, Montreal Neurological Institute (MNI) coordinates, left side = -30, -8 24, right side = 26, -8, 24) and the left and right lateral ventricles (average cluster size = 440 mm^3^, MNI coordinates, left side = -10, -14, 22, right side = 8, -14, 22).

Following preprocessing, brain images were transformed into a common anatomical template space as follows: the FSL *flirt* algorithm was used to calculate the rigid-body transform of the mean fMRI brain volume to the T1-weighted anatomical brain volume, and the affine warp of the T1-weighted anatomical brain volume for each participant to the MNI152 (Montreal Neurological Institute) template [35] for each participant. The net transformation matrices were applied to the fMRI imaging data, which were resampled at a resolution of 3 mm x 3 mm x 3 mm. Due to the variability in brain size and morphology amongst the older adults (80+ years-old), the anatomical images were all visually inspected after both segmentation and transformation steps, and manually adjusted in cases where errors were identified.

The first level of analysis on the preprocessed imaging data was completed at the individual participant level using the NPAIRS analysis framework [36]. The BOLD fMRI signal response was estimated using a general linear model (GLM) to fit each task contrast of interest after convolving the design matrix with a canonical double-gamma hemodynamic response function [37], thereby obtaining a map of voxel-wise beta coefficient estimates. This was done separately for each task (TMT-A and TMT-B) run on the three trials of interest (*i*.*e*. ignoring trial one), then the corresponding pair of beta coefficient maps were used to obtain reproducible, z-scored maps of reliable task-related activation for each participant using the split-half statistical procedure of Strother et al. [36]. The analyses were done separately for two task contrasts, one which isolated activation associated with performing both parts of the TMT (*i*.*e*. TMT-(A+B) minus fixation) and another which isolated the different neural demands of TMT-B in relation to TMT-A (TMT-(B-A)).

The initial group analysis identified mean group-level activation maps based on the z-scored individual participant maps, using a one-sample t-test to identify significant areas of group-related activation. Significance was reported at a nominal voxel-level threshold of *p*<0.005, with cluster-size thresholding to control for false positives (cluster size = 253 for TMT-(A+B)-fixation; cluster size = 227 for TMT-(B-A)). Cluster-size thresholding was performed using a non-parametric permutation approach. For each analysis, activation maps were thresholded at a nominal p = 0.005 level. To obtain a corresponding null distribution, labels were randomly permuted, the data was re-analyzed and the same voxel-level threshold (2000 iterations) was applied. From the resulting data, the minimum cluster size (c_min_) was obtained such that the cumulative probability p(c>c_min_) = 0.05, and subsequently retained all cluster of size c>c_min_. Additional regression analyses tested for an association between individual participant activation z-scores and covariates of interest, including age and SPL. This was done by calculating a t-statistic on the regression coefficient to identify brain regions with significant loadings, with thresholding and effect size reporting done as described above. The group-level analyses were performed separately for the TMT-(A+B)-fixation and TMT-(B-A) contrasts. The SPL used in the covariate analysis was an average of both the TMT-A SPL and TMT-B SPL for the TMT-(A+B)-fixation contrast, and was TMT-A SPL subtracted from TMT-B SPL for the TMT-(B-A) contrast. Given the novelty of the data and potential importance in guiding future age-related research, for non-significant findings an exploratory analysis was conducted at the previously established voxel-level threshold of p = 0.005, but a relaxed cluster-size threshold of c = 20.

Motion parameters were reported for all participants as the root-mean-square (RMS) of displacement in 3D space. This metric was correlated with age and performance on the TMT using Spearman’s correlation.

## Results

### Participants

Thirty-six participants were included in the final analysis. One participant was excluded from analysis due to an outlying MoCA score. Demographic and neuropsychological data for the participants are summarized in Table 1. The cohort had a median age of 78 years (inter-quartile range: 14.5 years); had similar proportions of females and males; and was well-educated. Participants performed similarly on neuropsychological testing with small interquartile ranges for the MoCA and TMT-A, but with more variability in TMT-B and TMT-(B-A) performance.

**Table 1:** Demographic and neuropsychological assessment scores of the group.

	Median (IQR)	Quartile 1	Quartile 3
**Age**	78.0 (14.5)	65.8	80.3
**Gender (female), N (%)**	20 (55.6%)		
**Years of education**	16.0 (3.3)	14.0	17.3
**MoCA score**	27.0 (2.0)	26.0	28.0
**Paper TMT-A**			
**SPL**	1.1 (0.35)	0.95	1.3
**N_E_**	0 (0)	0	0
**Completion Time (seconds)**	26.1 (8.3)	22.7	31.0
**Paper TMT-B**			
**SPL**	2.8 (1.7)	2.0	3.7
**N_E_**	0 (1.0)	0	1.0
**Completion Time (seconds)**	65.1 (39.4)	46.5	85.9
**Paper TMT-(B-A)**			
**SPL**	1.6 (1.4)	1.0	2.4
**N_E_**	0 (1.0)	0	1.0
**Completion Time (seconds)**	36.7 (32.0)	21.3	53.2

Values reported in median (interquartile range, IQR) format unless otherwise stated. *N*, number of participants; MoCA, Montreal Cognitive Assessment; TMT, Trail-Making Test; SPL, seconds per link; N_E_, number of errors.

### fMRI behavioural results

The results of the tablet-based TMT showed a statistically significant correlation between age and various behavioural metrics for TMT-A, TMT-B, the average of both (denoted TMT-(A+B)) and the difference between TMT-B and TMT-A (denoted TMT-(B-A); see Table 2). For TMT-A, there was a significant positive correlation between age and SPL (rho = 0.594, p < 0.001). For TMT-B, there was a significant positive correlation between age and SPL (rho = 0.612, p < 0.001) and N_E_ (rho = 0.404, p = 0.014). For TMT-(A+B), there was a significant positive correlation between age and SPL (rho = 0.647, p < 0.001) and N_E_ (rho = 0.360, p = 0.031). Similarly, for TMT-(B-A) there was a significant positive correlation between age and SPL (rho = 0.439, p = 0.007) and N_E_ (rho = 0.406, p = 0.014).

**Table 2:** Analysis of the effect of age on the performance on the tablet-based TMT.

	Median (IQR)	rho	*p-value*	95% CI	
				Lower	Upper
**TMT-A**					
**SPL**	2.0 (1.0)	0.594	< 0.001	0.331	0.772
**N_E_**	0 (0.08)	-0.134	0.43	-0.443	0.203
**TMT-B**					
**SPL**	3.2 (1.4)	0.612	< 0.001	0.355	0.782
**N_E_**	0.83 (1.1)	0.404	0.014	0.088	0.646
**TMT-(A+B)**					
**SPL**	2.6 (1.4)	0.647	< 0.001	0.405	0.804
**N_E_**	0.5 (0.5)	0.360	0.031	0.037	0.615
**TMT-(B-A)**					
**SPL**	1.1 (0.8)	0.439	0.007	0.130	0.670
**N_E_**	0.67 (1.3)	0.406	0.014	0.090	0.648

The data were averaged across three trials for each TMT-A and TMT-B and for all six trials of TMT-(A+B) together. Values are reported in median (interquartile range, IQR) format. Rho is the correlation coefficient for the correlation between performance on the TMT and age using Spearman’s rank correlation; *p*-values are reported from the Spearman’s rank correlation; CI is the confidence interval bounds of the correlation coefficient; TMT, Trail-Making Test; SPL, seconds per link; N_E_, number of errors.

In addition, the performance on the tablet-based TMT tasks was significantly correlated with the results observed on the paper versions. Both TMT-A and TMT-B had a significant positive correlation between SPL on the two versions of the task with moderate strength (TMT-A, rho = 0.486, p < 0.005; TMT-B, rho = 0.405, p = 0.015). However, there was a weak, non-significant correlation between the two task versions for SPL of TMT-(B-A) (rho = 0.142, p = 0.409).

There was a significant difference in task performance between the tablet and paper versions of the TMT. For both TMT-A and TMT-B, there was a significant difference in SPL (TMT-A, p < 0.001; TMT-B, p = 0.046). Meanwhile for TMT-(B-A), the difference in SPL was not significant (p = 0.143).

### fMRI results

**Fig 3:**
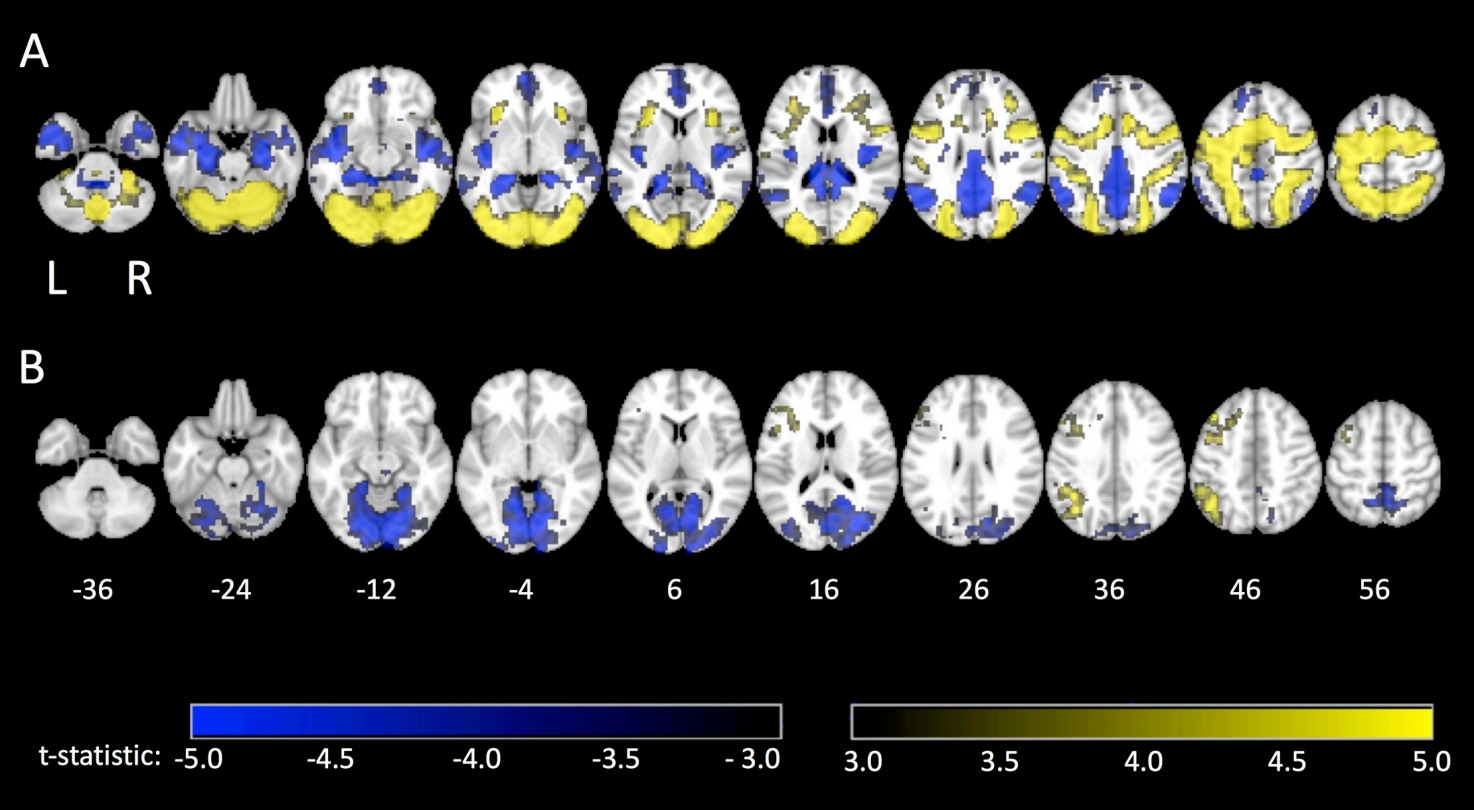
Brain activation maps during task completion. Group-level activation maps for tablet-based performance of (A) the TMT-(A+B) contrasted with fixation, and (B) TMT-B contrasted with TMT-A (*i*.*e*. TMT-(B-A)).

Fig 3A displays brain regions with significant group-level activation for the contrast of TMT-(A+B) with fixation, with significant clusters reported in Table 3. A spatially extensive pattern of positive activity was observed in the brain (*i*.*e*., increased activity during TMT-(A+B) compared to fixation), with positive activations seen in the visual cortex that extend into both the parietal lobes and upper cerebellum. Activity was also observed in prefrontal regions, centered mainly in regions of the inferior and middle frontal gyri, sensorimotor areas and the supplementary motor area, also encompassing subcortical areas including the insula, putamen and pallidum. Negative group-level activations (*i*.*e*. decreased activity during TMT-(A+B) compared to fixation) were also spatially extensive, concentrated predominately in areas of the posterior cingulate and angular gyri as well as the bilateral temporal poles, hippocampal formation and superior frontal lobe.

**Table 3:** Clusters of voxels showing significant group-level activation during performance of TMT-(A+B) contrasted with fixation and performance of TMT-(B-A).

Cluster Number	Cluster Size (voxels, mm^3^)	Center of Mass (x,y,z)	Peak Value (t-statistic)	Anatomical Region
TMT-(A+B) vs Fixation				
**1**	18,612	0, -48, 18	12.30	Left superior frontal gyrus
**2**	26,508	0, -42, 15	-12.95	Left posterior cingulate gyrus
**3**	1151	-3, 48, 18	-5.35	Left anterior cingulate gyrus
**TMT-(B-A)**				
**1**	5578	6, -72, 3	-7.71	Right lingual gyrus
**2**	631	-36, 18, 33	6.40	Left inferior frontal gyrus, pars opercularis
**3**	476	-42, -57, 42	5.70	Left angular gyrus

Spatial locations of the center of each cluster are reported in Montreal Neurological Institute (MNI) coordinates.

Fig 3B displays significant group-level brain activity associated with the TMT-(B-A) contrast, with significant clusters reported in Table 3. Positive activity (*i*.*e*., increased activity in TMT-B compared to TMT-A) was observed in various cortical areas, predominantly lateralized to the left hemisphere. Positive activation was observed in the left inferior parietal lobe, including the angular and supramarginal gyri, as well as the inferior, middle and superior gyri of the left frontal lobes. Additionally, the left insular cortex and middle cingulate gyrus exhibited significant clusters of positive activity. Negative activations (*i*.*e*., decreased activity in TMT-B compared to TMT-A) were focused in posterior brain regions, namely the bilateral cerebellum and occipital lobes, extending into areas of the middle temporal lobe and the precuneus of the parietal lobe.

**Fig 4:**
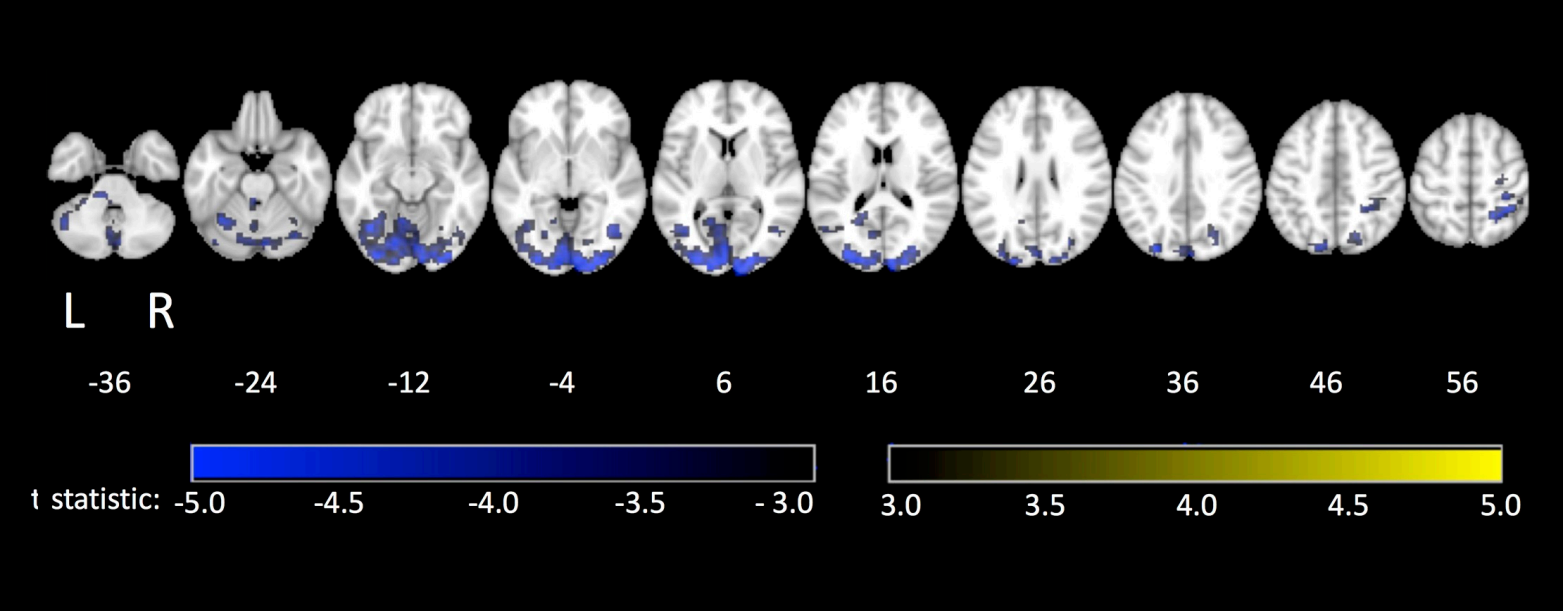
Brain activation maps covaried with age. Regions that show significant associations between age and brain activity for tablet-base performance of (A) TMT-(A+B) contrasted with fixation.

Fig 4 identifies brain areas that show significant effects of age on brain activity for the contrast of TMT-(A+B) with fixation, with significant clusters reported in Table 4. There was no significant positive association between brain activity and age. A distributed pattern of brain regions showed negative associations of brain activity with age, with large clusters in the bilateral occipital lobes, middle temporal lobes and cerebellum as well as small clusters in regions of the right parietal lobes, including the postcentral gyri and the inferior parietal lobe. There were no significant associations between age and brain activity in the TMT-(B-A) contrast.

**Table 4:** Clusters of voxels showing significant effects of with age during performance of TMT-(A+B) contrasted with fixation.

Cluster Number	Cluster Size (voxels, mm^3^)	Center of Mass (x,y,z)	Peak Value (t-statistic)	Anatomical Region
**TMT-(A+B) vs Fixation**				
**1**	3868	-3, -78, -3	-7.50	Left lingual gyrus
**2**	375	27, -45, 57	-5.34	Right postcentral gyrus
**3**	331	-27, -63, -48	-5.63	Left cerebellum

Spatial locations of the center of each cluster are reported in Montreal Neurological Institute (MNI) coordinates.

No brain regions had a statistically significant associations with our measure of task performance (SPL), for both contrasts (TMT-(A+B) vs fixation and TMT-(B-A)). A follow-up exploratory analysis was therefore completed at a relaxed cluster-size threshold (see Table 5 for cluster report). The results identified additional brain regions that had negative associations of brain activity with age in the TMT-(A+B) vs. fixation contrast, including bilateral regions of the thalamus and precentral gyrus, left lateralized regions of the parietal lobe (postcentral gyrus, superior parietal lobe), lingual gyrus and supplementary motor area and right lateralized areas of the frontal lobe (inferior and middle frontal gyrus) and supramarginal gyrus. Two clusters also showed positive associations of brain activity with age, centered in the right rectus and the right medial frontal gyrus (pars orbitalis). Several brain areas exhibited positive associations with age in the TMT-(B-A) contrast, including larger clusters located in the left cerebellum and occipital lobe and small clusters in the left temporal lobe, right insula and bilateral frontal lobes (left superior frontal gyrus, right middle frontal gyrus).

**Table 5:** Clusters of voxels from the exploratory analysis showing effects of age and SPL during performance of TMT-(A+B) contrasted with fixation and performance of TMT-(B-A).

Cluster Number	Cluster Size (voxels, mm^3^)	Center of Mass (x,y,z)	Peak Value (t-statistic)	Anatomical Region
**TMT-(A+B) vs Fixation; Covariate: Age**				
**1**	3868	-3, -78, -3	-7.50	Left lingual gyrus
**2**	375	27, -45, 57	-5.34	Right postcentral gyrus
**3**	331	-27, -63, -48	-5.63	Left cerebellum
**4**	319	-27, -45, 51	-7.71	Left superior parietal gyrus
**5**	140	9, 54, -15	4.89	Right rectus
**6**	112	18, -15, 3	-4.74	Right thalamus
**7**	98	-18, -15, 3	-4.12	Left thalamus
**8**	69	57, -24, 42	-4.27	Right supramarginal gyrus
**9**	54	57, 12, 27	-4.60	Right inferior frontal gyrus, pars opercularis
**10**	50	-33, 0, 57	-4.94	Left precentral gyrus
**11**	30	42, 0, 57	-5.14	Right middle frontal gyrus
**12**	29	-60, -18, 36	-4.19	Left postcentral gyrus
**13**	29	-57, 9, 33	-4.63	Left precentral gyrus
**14**	28	-9, 9, 69	-4.89	Left supplementary motor area
**15**	27	-39, 0, 39	-3.79	Left precentral gyrus
**16**	26	9, 30, -9	3.48	-
**17**	25	42, 0, 39	-4.10	Right precentral gyrus
**TMT-(B-A); Covariate: Age**				
**1**	224	-3, -69, -6	5.75	Vermis
**2**	27	-12, -90, 0	4.88	Left calcarine sulcus
**3**	23	-45, 3, -9	4.43	Left superior temporal gyrus
**4**	23	-6, 36, 51	3.82	Left medial superior frontal gyrus
**5**	21	42, 15, -12	4.12	Right insula
**6**	21	48, 18, 42	5.41	Right middle frontal gyrus
**7**	20	3, 27, 33	3.97	Right middle cingulate gyrus
**TMT-(A+B) vs Fixation; Covariate: SPL**				
**1**	38	-21, -18, 27	5.99	-
**2**	35	-3, -57, -54	-4.64	Left cerebellum
**3**	28	45, -30, -18	4.54	Right inferior temporal gyrus
**4**	27	-15, -78, 42	4.07	Left superior occipital gyrus
**5**	20	-12, 30, 45	-4.25	Left superior frontal gyrus
**TMT-(B-A); Covariate: SPL**				
**1**	176	6, -15, -27	-6.20	-
**2**	77	-21, -93, -21	-6.39	Left cerebellum crus
**3**	71	-21, -63, -30	-5.46	Left cerebellum crus
**4**	58	-51, -63, -18	-5.36	Left inferior temporal gyrus
**5**	50	-27, -27, -21	-5.36	Left parahippocampal gyrus
**6**	46	15, -90, -21	-8.76	Right cerebellum crus
**7**	46	12, 21, 57	4.20	Right supplementary motor area
**8**	39	-30, -48, -3	5.75	Left lingual gyrus
**9**	38	51, 36, -18	-5.40	Right inferior frontal gyrus, pars orbitalis
**10**	33	-9, 3, 12	5.56	Left caudate nucleus
**11**	25	-15, -57, 3	-3.82	Left lingual gyrus
**12**	25	-48, -30, 3	-4.00	Left middle temporal gyrus
**13**	23	-18, -33, -36	-4.78	-
**14**	21	-18, -54, -63	-4.85	-
**15**	21	-21, -90, -42	5.44	-
**16**	21	-24, -3, -36	-5.64	Left fusiform gyrus
**17**	21	9, 0, 15	6.09	Right thalamus
**18**	20	33, 39, 42	5.91	Right middle frontal gyrus

Thresholding was based on p = 0.005 voxel-wise thresholding with a relaxed cluster-size threshold of 20. Spatial locations of the center of each cluster are reported in Montreal Neurological Institute (MNI) coordinates.

In the exploratory analysis, performance (SPL) had both negative and positive associations with brain activity in the TMT-(A+B) vs. fixation contrast. Clusters of negative effects were centered on the left hemisphere in regions of the cerebellum and the superior frontal gyrus. Meanwhile, clusters of positive effects were located bilaterally in the right inferior temporal gyrus as well as the left caudate nucleus and left superior occipital gyrus.

Similarly, brain activity in the TMT-(B-A) contrast showed both negative and positive associations with performance (SPL). Negative effects were observed bilaterally in the cerebellum, parahippocampal gyri and inferior frontal gyri and left lateralized in the occipital and temporal lobe. Positive effects were centered in the bilateral caudate nuclei, the right frontal lobe and motor areas, and the left cerebellum and occipital lobe.

The average motion parameters across both fMRI scans (Run 1 and Run 2) are listed for each participant in S1 Table. Head motion showed a moderate, significant correlation with age (rho = 0.439, p = 0.007) and a weak, significant correlation with the performance metrics for TMT-B (SPL, rho = 0.334, p = 0.046; N_E_, rho = 0.331, p = 0.049).

## Discussion

This study was the first to investigate brain activity during TMT performance in a cohort of older adults, using novel fMRI-compatible tablet technology to administer a realistic replication of the TMT in an MRI environment. All participants went through extensive screening to ensure they were cognitively intact and the group median score on the MoCA was 27, which is classified as a normal, healthy score [30]. For the group, contrasting both conditions of the TMT (TMT-A and TMT-B) with fixation revealed extensive bilateral recruitment of brain areas in the frontal, parietal, temporal and occipital lobes and key motor areas common to both tasks. In comparison, contrasting TMT-B with TMT-A identified task-related activity in the frontal and parietal lobes that was largely lateralized to the left hemisphere. Within this group, aging was significantly associated with brain activity for the contrast of TMT-B and TMT-A with fixation. For this contrast, older age was significantly associated with an extensive decrease in brain activity, particularly in regions of the bilateral occipital, temporal and parietal lobes, as well as worse performance on the task.

The activation patterns found in this study are generally consistent with those found in previous imaging studies of the TMT for younger populations (~20–35 years old) [18,19,21,22,25,38,39]. There were some slight differences with other studies, which are elaborated on later in the discussion. In contrast to fixation, both TMT conditions were associated with activation of the bilateral frontal, parietal, temporal and occipital lobes. The frontal lobe and more specifically the prefrontal cortex, has been historically associated with the TMT due to its role in executive functions, such as attention, processing speed and planning, which are crucial for task coordination and completion [18,19,21,22,25,26,38–40]. The medial temporal lobe has also been linked to executive functions involved in the TMT [41–43]. In addition, the temporal lobe may be recruited due to working memory demands of recalling the numbers and letters during the TMT [21,22]. Both the occipital and parietal lobes have been implicated in TMT performance due to their involvement in visual search abilities [25,44,45]. Specifically, the parietal lobe aids in task completion through its role in visuospatial ability, which helps integrate visual information to guide movements in space [46,47].

Contrasting TMT-B to TMT-A revealed positive activation that was largely lateralized to the left hemisphere for the frontal and parietal lobes. Left lateralization of brain activity has been previously observed in neuroimaging studies of TMT-B compared to TMT-A [18,21,22,25]. However, in a study by Mueller et al., it was identified that TMT-B was found to evoke bilateral activity in prefrontal and premotor areas, with more activity observed in the right hemisphere [39]. Similarly, Shibuya-Tayoshi et al. found that the majority of their participants displayed bilateral patterns of activation in the prefrontal cortex during completion of TMT-B [26]. Both aforementioned studies used functional near-infrared spectroscopy (fNIRS) to measure brain activity, meanwhile studies employing fMRI, this study included, have observed a strong left hemisphere lateralization of brain activity associated with TMT-B. The different neuroimaging modalities and resulting methods of TMT delivery may contribute to the discrepancy in results between these studies.

Investigations of the TMT in younger populations have reported increased activity in the frontal, parietal and temporal lobes during TMT-B compared to TMT-A, consistent with what was found in this investigation [22,25]. As previously noted, these cortical areas have important cognitive functions for TMT completion and may be more active during TMT-B due to the increased complexity of this condition. Negative task-related activity in both contrasts was observed in areas involved in the default mode network (DMN), including the posterior cingulate cortex, precuneus, angular gyri, hippocampus, inferior parietal lobe and temporal lobe [48–50]. Previous research supported DMN activation during periods of wakeful rest [51] and DMN suppression during cognitive stimulation, including performance of complex tasks (e.g. TMT).

Two previous fNIRS studies also investigated brain activity associated with the TMT in an older population (50+ years old) and compared the results between young and old age groups [38,39]. Both observed more activated channels in the older group when contrasting TMT-B with TMT-A [38,39], similar to the results found in this study. However, as an imaging method, fNIRS has restricted spatial resolution and can only measure activity 1.5 cm deep into the cortex of the brain, which can limit precision when identifying activated brain areas [52]. Functional MRI is advantageous in comparison because activity is measured with millimetre spatial resolution throughout the entire brain volume, as appropriate for studying the TMT. For example, a recent study by Churchill et al. (2017) used an earlier version of the MRI-compatible tablet technology [22] to deliver a modified version of the TMT to young (21–33 years old) and older (61–82 years old) cohorts [53]. The older cohort showed less brain activity in posterior brain areas, supporting the results of the current investigation.

One limitation of these previous fNIRS and fMRI studies was that the experiments were designed to compare the average brain activity between two different age cohorts. Such an approach may not account for age-related variation in brain activity within each cohort, especially if the age-range within a given cohort is substantial; this has been previously observed for TMT-related brain networks, e.g., in (Churchill et al. (2017)). This effect was observed in the present study, which showed that there were performance and brain activity differences between participants at the younger end of the cohort (e.g. 60 years old) and those at the older end (e.g. 75 years and older). Thus, examining age-related variability in brain function as a continuous covariate may provide additional useful information.

The current study revealed extensive areas across the bilateral occipital, middle temporal and parietal lobes with reduced activity in older aged participants during both TMT conditions contrasted to fixation. Increased age was associated with longer time to complete links and more errors during the task, which is consistent with current literature on the age-related changes in TMT performance [13–16]. Previously it was suggested that cognitive deficits in processing speed, attention and mental flexibility may be affecting performance in healthy, elderly populations [15,16]. The current findings support existing literature, which identifies age-related cognitive slowing and decline in many domains including memory, spatial ability, attention, processing speed, reasoning and global cognition, irrespective of health and education levels [54–56]. This is in accordance with previous studies, which have revealed both performance decrements and reduced levels of brain activity to be associated with increased age in a cognitively healthy older adult population [57,58]. Importantly, the results of this study clarify the neural basis underlying specific age-related changes in task performance, since the identified brain areas are implicated in cognitive functions vital for the TMT. The occipital, temporal and parietal lobes have well-characterized age-related changes in structure and function, which affect cognitive abilities of the elderly population in many domains including attention, visual processing, executive function and working memory [59–67]. The medial temporal lobe in particular is of substantial interest aging studies, due to its role as a clinical biomarker for forms of dementia [41,68–73].

Contrasting TMT-B to TMT-A revealed non-significant effect when adjusted for multiple comparisons. However, a more relaxed threshold identified greatest effects on brain activity in areas of the left cerebellum and occipital lobe associated with increased age. Due to the increased difficulty of TMT-B, as demonstrated by the higher SPL values, greater cognitive resources are required to complete the task and this effect may be exacerbated in older adults who need to exert more cognitive effort for task completion. Older participants had higher activity in the occipital lobe, which has identified visual processing and visual search functions, and would be necessary for localizing numbers and letters throughout task completion. The cerebellum has been identified as important for executive functions, particularly involving multitasking, working memory and divided attention [74–76], which would be increasingly important for TMT-B completion as it requires switching between numbers and letters. Behaviourally, older age was significantly associated with increased errors and slower completion (SPL during TMT-B). This suggests that despite increased brain activity during TMT-B, older participants tend to perform worse on the task. The age-related effect on brain activity was more extensive for the TMT-(A+B) vs fixation contrast. This may be because the TMT-(B-A) contrast has more focal patterns of activation, therefore there are fewer brain areas where an aging affect can be observed.

Sparse patterns of activation were correlated with task performance as measured by SPL of TMT-(B-A). When contrasting TMT-B with TMT-A, decreased activity in the left inferior occipital lobes and bilateral cerebellum was associated with worse performance (*i*.*e*. increased SPL). As previously discussed, the occipital lobes and cerebellum have significant cognitive functions that are involved in task completion. Research studies have established that increased neural efficiency is associated with better cognitive performance [77–79], suggesting that skilled participants have more efficient brain functioning leading to decreased brain activity and recruitment of less brain regions.

The results of this study reveal the neural networks underlying TMT performance in a healthy older adult population–a clinically relevant control population due to the widespread use of the TMT as a screening tool for dementia. With the present results serving as the foundation, future fMRI research should investigate brain activity during TMT performance in patients with mild cognitive impairment (MCI) as well as those with early probable AD. Such research could provide valuable information on TMT-related brain activity in the full spectrum of cognitive impairment, and on how the associated activation patterns relate to the observed deficits in task performance. In addition, such research will help to inform clinicians on the neuropathology detected by the TMT and may potentially reveal new combined behavioural and neuroimaging biomarkers that characterize impaired TMT performance.

The current study uses novel technology to replicate a cognitive test environment using the best methods to date. However, it is not an exact replication with a few limitations to be noted, including the supine position of participants, the limited field of view and restricted task block lengths. Participants were allotted 60 seconds to complete TMT-B to align with a block-design fMRI experiment, which focuses on networks that are most consistently engaged across participants and over time. This design may limit the ability to observe neurocognitive processes, which emerge later in the TMT task specifically among individuals who complete the task slower. More detailed future analyses should be conducted to identify transient components of the task with a different task block design. Additionally, the tablet may have added difficulty to completion of the TMT as shown by the significant difference in SPL between tablet and paper versions of the TMT. Although there was a significant correlation in SPL between the two versions for TMT-A and TMT-B, there was no significant correlation for TMT-(B-A). This is a potential limitation of the tablet version of the TMT and should be addressed in future studies to ensure it is more consistent with the traditional paper version.

As a final note, it is important to recognize that the physiological effects of aging on the BOLD fMRI signal are a potential confound to interpreting the study findings [80]. Future studies should incorporate additional physiological imaging measures, such as BOLD fMRI of cerebrovascular reactivity during a breath-hold task, to control for neurovascular coupling confounds [81] and confirm results found in this study. Notwithstanding this recommendation, the present results are in good agreement with prior research investigating the brain activation patterns underlying the TMT, suggesting that the age-related physiological confounds have not significantly impacted the results of this study.

## Conclusions

Using novel tablet technology and fMRI, this was the first study to investigate neural areas recruited during a realistic version of the TMT in an older adult population (50+ years-old). Across the older aging group, both conditions of the TMT recruited activity from extensive brain regions across the bilateral frontal, parietal, temporal and occipital lobes. Older age had an impact on brain activity and function during both TMT conditions, such that older age was associated with reduced brain activity and worsened task performance. In particular, there was decreased task-related activity in the medial temporal lobe, which is a common clinical biomarker for dementia. This finding is important as it provides additional support for the use of the TMT as a clinical screening tool for dementia. Future studies are necessary to determine the changes in brain activity patterns in cohorts with diagnosed cognitive impairment along the spectrum from mild cases (MCI) to more severe cases (AD). This will allow us to determine the difference between task-related neural networks employed in a healthy aging and a cognitively impaired population, providing insight into the neuropathology underlying impaired TMT performance and the efficacy of the TMT as a clinical screening tool.

## Supporting information

S1 Table The average motion parameters for each participant. Motion parameters for each participant averaged across run 1 and 2.
(DOCX)
