# Supplementary material for: Functional magnetic resonance imaging of the trail-making test in older adults
Source: PLoS One. 2020 May 12;15(5):e0232469. doi: 10.1371/journal.pone.0232469 (PMC7217471; doi:10.1371/journal.pone.0232469)
Supplement: S1 Table — Motion parameters for each participant averaged across run 1 and 2. (DOCX) [file pone.0232469.s001.docx]

| Participant Number | Average Absolute RMS (mm) |
| --- | --- |
| 1 | 0.23 |
| 2 | 0.29 |
| 3 | 0.24 |
| 4 | 0.26 |
| 5 | 0.50 |
| 6 | 0.17 |
| 7 | 0.24 |
| 8 | 0.22 |
| 9 | 0.31 |
| 10 | 0.46 |
| 11 | 0.68 |
| 12 | 0.33 |
| 13 | 0.39 |
| 14 | 0.29 |
| 15 | 0.36 |
| 16 | 0.41 |
| 17 | 0.33 |
| 18 | 0.13 |
| 19 | 0.16 |
| 20 | 0.48 |
| 21 | 0.52 |
| 22 | 0.26 |
| 23 | 0.40 |
| 24 | 0.54 |
| 25 | 0.53 |
| 26 | 0.37 |
| 27 | 0.61 |
| 28 | 0.34 |
| 29 | 0.90 |
| 30 | 0.29 |
| 31 | 0.45 |
| 32 | 0.44 |
| 33 | 0.41 |
| 34 | 0.76 |
| 35 | 0.45 |
| 36 | 0.35 |
